# Impact of ambient air pollution on outdoor employees’ performance: Mediating role of anxiety

**DOI:** 10.3389/fpsyg.2022.926534

**Published:** 2022-09-28

**Authors:** Muhammad Waseem Bari, Shaham Saleem, Mohsin Bashir, Bashir Ahmad

**Affiliations:** ^1^Lyallpur Business School, Government College University, Faisalabad, Pakistan; ^2^School of Management and Economics, Beijing Institute of Technology, Beijing, China; ^3^Department of Public Administration, Government College University, Faisalabad, Pakistan

**Keywords:** ambient air pollution, processing efficiency theory, anxiety, employee performance, pharmaceutical industry

## Abstract

This paper aims to examine the direct and indirect impact of ambient air pollution (AAP) on employees’ performance. This study has used cross sectional survey design to collect the data from the outdoor employees of the pharmaceutical industry of Pakistan. The data were collected in time lags from 299. Partial least squares- structural equation modeling (PLS-SEM) approach was applied to analyze the data. The results show that AAP has a significant negative impact on the employees’ performance, and anxiety partially mediates the association between AAP and employees’ performance. This study reveals that AAP brings anxiety among outdoor employees, which in turn decreases their working performance. The implications, limitations, and future research directions are presented in the last section of this study.

## Introduction

Ambient air pollution (AAP), a composition of various harmful gases and particles (Mannucci and Franchini, 2017; Abera et al., 2021), has become a major environmental problem that human being is facing currently. According to the World Health Organization (WHO), AAP was responsible for approximately seven million deaths in 2012 (WHO, 2014; Requia et al., 2018), with most of these death were related to ischemic heart disease (40%), stroke (40%), chronic obstructive pulmonary disease (11%), lung cancer (6%), and acute lower respiratory infections (3%) (WHO, 2014; Elder et al., 2016; Mannucci and Franchini, 2017). In addition to this, AAP also decreases mental performance (Zhang et al., 2018) and sleep (Heyes and Zhu, 2019), and increases depression (Fan et al., 2019) and health expenditure (Pu et al., 2021). Recently, researchers have started to examine the new aspects of AAP including its effect on worker productivity (Zivin and Neidell, 2012), safety performance (Ahmadi et al., 2021), cognitive performance (Zhang et al., 2018), human performance (Mullins, 2018), and trade performance (Huang et al., 2020). Empirical evidence indicates that AAP negatively influences the individual and organization-level outcomes. Therefore, organizational scholars in recent years are paying increased attention to the impacts of AAP in order to find the ways to curb it devastating effects (Zhang et al., 2018; Ravindra et al., 2021).

This study focuses on the impact of AAP on the performance of outdoor employees. These employees perform their duties outside the organizations such as sales personnel. They are important organizational members, and their performance is important for organizational effectiveness. Since they spend most of their time outside the organizations, therefore they are more likely to influence by the AAP. For example, Graff Zivin and Neidell (2012) found that air quality standards significantly influence productivity agriculture worker. In another study, Archsmith et al. (2018) examined the impact of air pollution on the work performance of a high-skilled group that was composed of quality-focused individuals. However, very little research has empirically explored why and how AAP can impact the outdoor employees’ performance. This is an important omission in the literature, and highlight the need for further research into this important research stream (McCrink-Goode, 2014; Yeganeh et al., 2018; Fan et al., 2019).

The main aim of this study is to examine the relationship between AAP and outdoor employees’ job performance. In addition, this study also intends to explore the underlying mechanism through which AAP influences job performance. This study takes anxiety, a state in which an individual is unable to instigate a clear pattern of behavior to remove or alter the event/object/interpretation that is threatening and existing goal (Power and Dalgleish, 1997; Eysenck et al., 2007; Derakshan and Eysenck, 2009), as a potential mediator between AAP-job performance nexus.

Past literature shows that pollution (AAP) significantly increases the depression and anxiety (Ivandic et al., 2017; Pun et al., 2017a). Anxiety, basically, is the state of the aversive motivation of an individual that prevails in a situation in which the perceived threat is high. When this threat in the form of AAP is high, it causes anxiety, which in turn diminishes the work performance (Calvo and Eysenck, 1992). Several studies have concluded that anxiety makes a job more stressful and reduces job satisfaction (Newbury-Birch and Kamali, 2001; Melchior et al., 2007; Wongtongkam et al., 2017). Employees with anxiety have complications at work, which in turn harm their performance (Jones et al., 2016).

Like other South Asian countries, Pakistan is also facing the problem of AAP, especially in the big cities, including Karachi, Lahore, and Faisalabad. The air quality of these cities has become worse due to industrialization, high traffic, and high-level of burning of fossil fuel (Alvi et al., 2018; Hashmi et al., 2018; Hamid et al., 2019). The high level of AAP is significantly impacting the psychological and physical wellbeing of people (Mehmood et al., 2018). Though Pakistan is taking several measures to control AAP and its effects, but the success rate of these measures is very low. Resultantly, the AAP is not only affecting the general public, but also the work class such as outdoor employee (i.e., sales and marketing staff). This study has particularly focused the pharmaceutical industry of Pakistan that hires thousands of staff for sales and marketing. They are named as medical representatives, and are active 24/7 usually on their bikes in the market to achieve their job targets. Considering the nature of their job, they are more likely to be negatively affected by the AAP.

Hence, this research intends to explore the impact of AAP on the job performance of medical representatives from pharmaceutical industry in Pakistan. The objective of this study is twofold. First, to investigate the impact of AAP on outdoor employees’ job performance Second, to examine anxiety as an outcome of AAP, and how it mediates AAP-performance link. This study contributes to existing literature in two important ways. First, it contributes to the literature on air pollution and its impact on job related outcomes in the organizational context. Second, it contributes to the literature by exploring the psychological mechanism through which AAP impacts job performance of outdoor employees.

The remainder of this paper is organized as follows: literature review, methodology, results and analyses, discussion, study implications (theoretical and managerial), limitation, and future research directions.

## Literature review

The twenty-first century realized to the corporate world that environmental stability is an important factor for their sustainable growth. In modern economies, a plethora of quantitative and qualitative studies exist which have been undertaken the impact of different environmental conditions on the worker’s performance, particularly in cognitive and physically demanding occupations. The scholars claim the impact of pollution exposure on cognitive performance during their pollution-productivity nexus and it disrupts employees’ performance (Dominski et al., 2021). For instance, Roth (unpublished)^1^ argued that mental health is indispensable to productivity in all professions, and it has a possible direct link with air quality. Ebenstein et al. (2016) investigated the association between pollution and student’s cognitive performance during exams and concluded that pollution exposure (PM_2.5_) has a significant impact on the students’ performance. Similarly, Zhang et al. (2018) examined the impact of air pollution on cognition scores of math and verbal tests and confirmed that the impact of AAP is more serious than verbal tests.

A systematic literature review conducted by Yeganeh et al. (2018) concluded that AAP (i.e., ambient air temperature) significantly decreases the cognitive performance of the individuals. Sunyer et al. (2017) explored the association between air pollution (traffic-related) and individuals’ activeness based on the daily variation of air pollution in Spain. Findings revealed that daily ambient levels of air pollution (elemental carbon and nitrogen dioxide) and individuals’ activeness are negatively correlated. Sunyer et al. (2017) further revealed that AAP has harmful effects on neurodevelopment. A meta-analysis on “air pollution and cognitive functioning across the life course” posits that there is a significant impact of traffic-related pollution on quantifiable impairment of brain development in young and cognitive decline in elder (Clifford et al., 2016). Based on the empirical evidence, Clifford et al. (2016) concluded that there is a limited number of studies that include the impact of different pollution indices on neurodevelopment. That is why the current study emphasizes the impact of air pollution on the employee’s job performance which eventually affects their productivity (Ming et al., 2022).

Chang et al. (2019) conducted interesting research by using the data of two Chinese call centers situated in Shanghai and Nantong. The study outcomes revealed that 10 points increase in the AAP index decrease the performance of workers by 0.35%. Likewise, Zivin and Neidell (2012) investigate the impact of Ozone pollution on agricultural worker’s productivity in the Central Valley of California and found that the Ozone level has a significant impact (i.e., 10 ppb average change in ozone exposure will change 4.2% of worker’s productivity) on the productivity of the agriculture workers (Chang et al., 2016). Archsmith et al. (2018) investigated the relation between air quality and error quantity in the largest cities of the United States by focusing on a specific group of employees (i.e., quality-focused and highly skilled) and confirmed the significant negative impact of fine Particulate Matter (PM_2.5_) and ambient carbon monoxide (CO) on the performance of this group.

Contrary, Zhan et al. (2018) hypothesized a relationship between green and lean environmental practices and economic growth (*via* high productivity). Post investigation on 172 Chinese manufacturing firms, the authors found that to gain environmental competitiveness, firms need to augment environmental awareness to their employees. Mulville et al. (2016) investigated the relationship between ambient environment and productivity in open-plan commercial offices and recommended that ambient environments significantly affect the health, comfort, and wellbeing which eventually influence the productivity of the workers. Another study on creative and productive workplaces concluded that environmental elements significantly affect the employee’s wellbeing which eventually influences their effectiveness and performance (Clements-Croome, 2015; Lee et al., 2022). It is an enormous reality that a poor environment contributes to absenteeism and workers cannot work well.

Pollution also affects the working capacity of employees which eventually affects the overall performance of an organization. A study in Mexico revealed that a 20% decrease in SO_2_ (air pollution) results in about a 3.5% (1.3 h) increase in working hours in the following week (Hanna and Oliva, 2015). Similarly, Fu et al. (unpublished)^2^ analyzed the reverse causality effect between air pollution (PM_2.5_ and SO_2_) and short-run labor productivity in China during 1998–2007 and recommended that improving air quality within organizations can enjoy substantial output and productivity benefits. The empirical evidence of this study demonstrated that 1 μ*g*/*m*^3^decrease in PM_2.5_ and SO_2_ increases labor productivity in manufacturing firms by 0.0084 and 0.0572%, respectively.

One of the personnel characteristics is the individual’s psychology. Psychological characteristics are less frequently found in environment-productivity nexus. However, according to Nakata et al. (2004) psychological characteristics (i.e., work stress) are directly linked with psychological reactions (i.e., insomnia, depressive symptoms, and job dissatisfaction). Another study investigated the impact of environmental factors on worker’s performance and their psychological characteristics (Realyvásquez et al., 2016; Li and Li, 2022). The findings revealed that there is an impact of environmental elements on the psychological characteristics of the employees. Moreover, environmental quality and employee performance have a significant positive relationship. In an organization, stress has a significant influence on employee’s physiological, psychological, and performance base elements (Prasad et al., 2015).

The literature on AAP demonstrates that employees’ physical health as well as their psychological health both harmfully influenced by AAP. The literature also sheds the light on work performance of employees and found that AAP affects unfavorably to task performance ability of workers (Pun et al., 2017b; Archsmith et al., 2018). Based on the above theoretical claims, the present study posits that AAP and employees’ outdoor performance are negatively associated. Hence, this study hypothesizes that:

Hypothesis 1: *AAP negatively influences outdoor employees’ performance*.

### Anxiety as a mediator

In the perspective of industrial and organizational psychology, anxiety is one of the essential mental illness that has a greater deal of attention s (Miller and Monge, 1985; Bowe et al., 2021). Dowsett et al. (2020) defined anxiety as a mental disorder that may take up from one’s childhood or adolescence. McNally (2002) categorizes “anxiety sensitivity” and “trait anxiety” are two different dimensions of anxiety. The trait anxiety refers to the general propensity of someone to respond to stressors fearfully, while anxiety sensitivity refers to the specific tendency of someone to respond to the symptoms of anxiety (McNally, 2002). Environmental and genetic issues are possible reasons for anxiety (Dowsett et al., 2020). Hettema et al. (2005) explained the elements in one’s surroundings like job stress, study-related problems, relationship with the spouse, and financial issues are possible reasons for anxiety. The anxiety disorder can be transformed through genes which are called genetic factors of anxiety. However, McNally (2002) noticed that only 45% of anxiety cases are caused by genetic factors of anxiety. Similarly, Brunst et al. (2019) said that “family history, socioeconomic status, and medical conditions” can also lead to anxiety. The employees’ emotional distress may be in form of poor task performance, absenteeism, job dissatisfaction, and burnout (McCraty et al., 2003). The dissatisfaction with the job can be caused by environmental issues.

According to the processing efficiency theory, worry impairs the effectiveness and efficiency of work and leads to a mental disorder that causes anxiety (Calvo and Eysenck, 1992). In this era of competition, exposure to AAP is the greatest environmental challenge faced by human beings (Miller et al., 2019). According to Vert et al., 2017a,b arguments, employees’ mental health can be potentially affected by air pollution. Pun et al. (2017b) revealed that pollution is a considerable cause of mental illness and stress. Brunst et al. (2019) also acknowledged that anxiety is considered one of the common mental disorders that occurred due to air pollution. Cognitive function (human’s ability to process) is also affected by air pollution (Brunst et al., 2019). Anger, discomfort, and irritability can also consequence of air pollution (Zeidner and Shechter, 1988). Zhao et al. (2020) also understand that AAP is a significant cause of anxiety and depression. Zeidner and Shechter (1988) extended this argument and explain that AAP not only dangerous for health but also has consequences, such as “soiling, corrosion, ecological impacts, and aesthetic damages.” The potentially harmful impact of AAP on health is cardiovascular disorder and respiratory-related infection.

Presbitero (2020) described that anxiety affects the task performance ability of employees. The mental health of employees has a positive association with their performance and productivity (McCraty et al., 2003). The employee’s emotional wellbeing is an important contributing factor for overall organizational achievements. McCraty et al. (2003) acknowledged that the positive attitude of employees can play a role as a determinant factor for the high profitability of the organization. Employees’ health and working conditions have a strong association with each other (Jones et al., 2016). Employees with anxiety disorder have more chances of poor task performance or quit from work (Plaisier et al., 2012). The anxiety may develop negative behaviors among employees such as presenteeism, absenteeism, turnover intentions, and low productivity. Contrary, employees’ psychological wellbeing may influence their task performance (Jones et al., 2016).

Research on the relationship between anxiety and employees’ work performance has generally found that anxiety and performance have a negative association with each other (Plaisier et al., 2012; Jones et al., 2016). AAP adversely affects human beings’ physiological and psychological perspectives. This study objective not only to know how anxiety impairs outdoor employees’ performance but also its mediating role between AAP and employee performance. Zeidner and Shechter (1988) point out that air pollution is intervened by different health reactions (e.g., discomfort, anxiety, depression). Hence, the present study proposed that;

Hypothesis 2: *Anxiety mediates the relationship between AAP and employees’ performance.*

Summary of previous research.

**TABLE:** 

Author(s)	Variables of study
Hinchliffe (2000)	Performance, experimental knowledge, outdoor management training
Williams et al. (2003)	Outdoor experiential training for leadership and team building.
Núñez et al. (2018)	Management skills, experiential learning, outdoor training, mindfulness.
Ke et al. (2021)	City air pollution, attitudes, subjective wellbeing.
	

**FIGURE 1:**
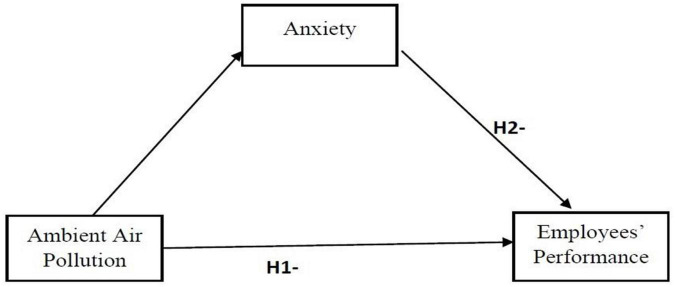
Research framework.

Above literature indicates that impact of air pollution on employee behavior is attracting the attention of organizational scholars, but very limited research has particularly investigate the role of AAP on outdoor employees’ performance by considering anxiety as an underlying mechanism. This is an important gap in the literature, thereby this study aims to fill this gap. Figure 1 presents the research framework of the study.

## Methodology

### Sample and data a collection procedure

The drug regulatory authority of Pakistan (DRAP) is a government institution. DRAP is “mandated for effective coordination and enforcement of the Drugs Act, 1976 to regulate, manufacture, import, and export, storage, distribution and sale of therapeutic goods in the country” (DRAP, 2020). As per the DRAP record, 6,020 pharmaceutical firms are operating across Pakistan (DRAP, 2020). The authors randomly selected 60 firms from the data list of DRAP. Although, the authors regularly approached these 60 firms through emails and telephonic calls, however, only 22 firms replied and showed their interest and agreed to participate in this research project.

The respondents’ desirability biases are controlled with the following procedure. With an administrative survey approach, the authors visited the human resource management/admin offices of the 22 firms and explained the objectives of this study. After getting the permission and consent of the firm owners/managers, the authors also met the employees and got their consent to complete the survey. The questionnaire was arranged in English with a cover letter that described the study objectives and committed the participants about data confidentiality. The cover letter also explained that only collective data would be used for analysis and individual recognition will not be shared. For rational answers, a request was also made to the participants that please stay relax during filling the questionnaire because there are no right or wrong answers. The approval of this study was taken from the institutional ethics committee and board of studies. The above-mentioned measures helped the authors to decrease social desirability/acquiescence biases from the data (Spector, 2006; Belso-Martinez et al., 2013; Meng and Bari, 2019; Cappa et al., 2020).

This study also concentrated on the potential of non-response biases. The authors examined this problem by comparing early and late responses (Dillman, 2011; Meng and Bari, 2019). A post-comparison revealed that there was no significant variance between the early and late responses from the participants. The data were gathered with the willingness and consent of the participants, without any social/professional pressure. The process of data gathering was completed in three waves (Time-1, Time-2, and Time-3) with a gap of 45 days each. To avoid the shuffling of different waves of data, the authors allotted a unique recognition number to each response form. In time-1, the questions about AAP and demographics of participants (i.e., experience, gender, education, and age) were asked. In time-2, the questions about anxiety were asked. In time-3, the questions about employee performance were placed. A total of 600 questionnaires were distributed to 22 pharma companies. The participants’ attrition rate during the three waves is explained in Table 1.

**TABLE 1:** Data attrition rate.

Time lags	Survey sections	Questionnaire distributed	Questionnaire returned	Questionnaire lost	Attrition rate (%)
Wave-1	AAP* and participants’ demographics	600	435	165	27.5
Wave-2	Anxiety	435	356	79	18.16
Wave-3	Employees performance	356	299	57	16.01

*Ambient air pollution.

The findings show that the data is reliable and can be used for further analysis. The measure of spread indicates that the sample of this study (299) appropriately represents the population of this research. Besides the time lags data approach, the present study also checked the common method bias by applying the “Harman’s single-factor test.” This is a *post hoc* approach to find out whether a single set factor is the cause of employees’ performance in the data set (Tehseen et al., 2017). This study performed “Harman’s single-factor test” through SPSS 21. The calculated results (principal axis factoring and extraction method) explained 15 dissimilar factors. The 1st unrotated factor took just 42.3% of the variance from the data. Hence, the two assumptions did not fulfill. First, the 1st factor did not cover the utmost of the variance (less than 50%). Second, no single factor appeared from the data set. Thus, the results confirm that common method variance is not the issue in this paper (Tehseen et al., 2017; Meng and Bari, 2019).

### Respondents’ profile

The profile of 299 respondents such as gender, marital status, age, experience, and education are mentioned in Table 2.

**TABLE 2:** Respondent’s demographic information.

Categories	Subcategories	Size	Percentage
			
Gender	Male	215	71.9
	Female	84	28.1
Marital status	Unmarried	128	42.8
	Married	171	57.2
Age	Less than 30	128	42.8
	31–35	98	32.8
	36–40	50	16.7
	41–45	21	7
	46 and above	2	0.7
Qualification	Intermediate	9	3
	Bachelor	36	12
	Master	101	33.8
	M.S	136	45.5
	PhD	10	3.3
	Others	7	2.3
Experience in years	Less than 3	72	24.1
	3–5	105	35.1
	6–10	65	21.7
	11 and above	57	19.1

### Measurements

#### Ambient air pollution

Following previous studies (i.e., Liao et al., 2015; Peng et al., 2019; Maione et al., 2021), this study also measured the perception of individual about air pollution. The AAP was measured by adopting the scale from a belief in the Global Warming Index Heath and Gifford (2006) and used by Gu et al. (2015). The five-point Likert scale was used to measure the AAP ranging from 1 (Strongly disagree) to 5 (Strongly agree). The sample item is “*It seems to me that air quality is worse now than in years before.”* The alpha value is 0.883.

#### Anxiety

*A*nxiety is measured with four items scale developed by Abramis (1994). The items are measured on a five-point Likert scale ranging from 1 (Not at all) to 5 (Extremely depression). The sample item is *“Nervousness or shakiness?.”* The alpha value is 0.887.

#### Employee performance

The employee performance is measured with the four items scale developed by Abramis (1994). The items are measured on a five-point Likert scale ranging from 1 (Very poor) to 5 (Exceptionally well). The sample item is *“Handling the responsibilities and daily demands of your work.”* The alpha value is 0.757.

### Statistical model

The present study applied partial least squares structural equation modeling (PLS-SEM) approach for variance-based methods regardless of co-variance-based methods like AMOS and LISREL. The selection of PLS-SEM is based on its effectiveness and equal reliability for confirmatory and exploratory studies (Hair et al., 2016). There are two types of structural equation modeling (SEM) Covariance-based (CB-SEM) and PLS-SEM (Hair et al., 2014). The key purpose of CB-SEM is to approve/disapprove theories, on the other hand, PLS-SEM is mainly used for the development, evaluation, and extension of theories (Hair et al., 2016; Bari et al., 2019a). The process of PLS-SEM analysis is passed through two stages, measurement model and structural model evaluation (Hair et al., 2014). The reliability of PLS-SEM is known for a complex and multi-orders-based model even for small data analysis (Bari et al., 2019b). It is also useful for decreasing biases during parameter estimates (Hair et al., 2016; Meng and Bari, 2019). This study analyzed the data through the latest version of SmartPls 3.9.

Comparison between Variance based-SEM and Covariance based-SEM

**TABLE:** 

Variance based SEM (PLS)	Covariance based SEM (AMOS)
Based on flexible theory and data driven	Based on strong theory and theory driven.
Requires small sample size	Requires large sample size
Relaxes assumption of normal distribution	Follows strictly assumption of normal distribution
Accepts both reflective and formative indicators	Generally considers reflective indicators
	

## Results and analysis

### Model measurement

The model fitness results show that the values fit indices meet the threshold criteria (SRMR = 0.063; d_ULS = 0.473; d_G = 0.174; Chi-Square = 312.974; NFI = 0.866). This suggests that our model is fit to data. The model of the present study comprises three variables and their 15 items. The model reliability is computed through Cronbach’s alpha (Hair et al., 2016). The experts recommended that the value of Cronbach’s alpha ≥ 0.7 shows a good level of model reliability (Hair et al., 2014). Table 3 explains that all variables Cronbach’s alpha values are ≥ 0.7. Composite reliability (CR), average variance extract (AVE), and each item reliability (factor loadings) techniques are applied to measure the convergent validity of the model (Hair et al., 2016). As per experts’ opinion, CR and AVE values of each variable should be > 0.7 and > 0.5, respectively. Table 3, the values of CR and AVE are considerably higher than the recommended threshold level. The factor loading/outer loading values of each item are also equal to or greater than 0.7 (Hair et al., 2016) as mentioned in Table 3.

**TABLE 3:** Model measurement.

Variables	Items	FLVs	CR	α	AVE
AAP	AAP1	0.715	0.909	0.883	0.588
	AAP2	0.793			
	AAP3	0.835			
	AAP4	0.775			
	AAP5	0.714			
	AAP6	0.800			
	AAP7	0.727			
Anxiety	ANTY1	0.842	0.922	0.887	0.747
	ANTY2	0.847			
	ANTY3	0.885			
	ANTY4	0.882			
Employee performance	EP1	0.771	0.845	0.757	0.577
	EP2	0.750			
	EP3	0.740			
	EP4	0.778			

CR, composite reliability; α, Cronbach’s alpha; AVE, average variance extracted; AAP, Ambient air pollution.

The authors evaluated the discriminant validity of the model by applying two known approaches, Fornell-Larcker criterion and heterotrait-monotrait (HTMT) ratios (Hair et al., 2016). According to the criterion of Fornell and Larcker (1981), the present study computed the square root of AVE of each variable and found the first value in every column higher than the other values in the same column. Table 4 Therefore, it is confirmed that this study model’s discriminant validity has been established (Fornell and Larcker, 1981; Hair et al., 2016). According to the HTMT ratios criterion, the HTMT ratio’s value should be < 0.85; however, the acceptable threshold is up to 0.90 (Hair et al., 2016; Bari et al., 2019a). Table 4 explains that all values of HTMT ratios are according to the given standard and confirm the discriminant validity existence in the present study.

**TABLE 4:** Discriminant validity.

Fornell–Larcker criterion					Heterotrait–monotrait (HTMT) ratios		
	AAP	Anty	EP		AAP	Anty	EP
AAP	0.767			AAP			
Anty	0.728	0.864		Anty	0.816		
EP	–0.428	–0.477	0.76	EP	0.513	0.575	

AAP, Ambient air pollution; Anty, anxiety; EP, employee performance.

The collinearity issues are also considered during analyses by observing the variance inflation factor (VIF). As per experts’ opinion, if the inner values of VIF are less than 5, it indicates, data are free from collinearity issues (Hair et al., 2014). The findings of this study confirmed that the variables’ inner VIF values are between 1.400 and 2.826. Hence, there are no collinearity issues in the data (Hair et al., 2016; Meng and Bari, 2019). The *R*^2^ value > 0.5 is considered a substantial model, particularly in primary data analyses. The *R*^2^ values of this model are 0.5 or near to 0.5, which is a sign of a good model (Hair et al., 2016). Moreover, the values of *Q*^2^ (cross-validated redundancy) of all three latent variables are higher than zero, which is another signal of a good model (Hair et al., 2016, 2014).

### Hypotheses analysis (direct effect)

**FIGURE 2:**
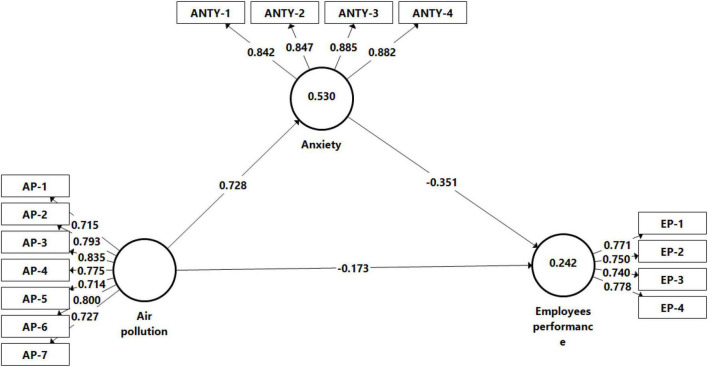
Post analyses model.

This study applied a bootstrapping technique, 4,500 samples with replacement to examine the significance level of hypotheses (Hair et al., 2016). This study investigated the effect of AAP on employee performance. Table 5, At 95% confidence interval, AAP has a significant negative association with employees’ performance (β = 0.173, *p* < 0.018). Thus, hypothesis 1 of this study is accepted. Figure 2 represents the output of the analysis.

**TABLE 5:** Direct relationship.

Structural paths	Path co-efficient (*t*-value)	Confidence interval (95%)	f^2^ effect size	*P*-values	Results
AAP → EP	–0.173 (2.374)	(–0.321 –0.035)	0.018	0.018	H1, accepted
AAP → Anty	0.728 (23.238)	(0.665 0.787)	1.127	0.000	
Anty → EP	–0.351 (4.733)	(–0.494 –0.201)	0.077	0.000	

AAP, Ambient air pollution; Anty, anxiety; EP, employee performance.

### Hypotheses analysis (mediation role)

To examine the mediation role of anxiety between air pollution and employee performance this study used the variance accounted for (VAF) technique (Bari et al., 2019a). According to this technique the VAF value > 80% represents full mediation, 20% < VAF > 80% represent partial mediation, and < 20% VAF value represent no mediation (Meng and Bari, 2019). The outcomes of this study show the VAF value is 59.81%. It indicates that anxiety partially mediated the relationship between AAP and employee performance. Thus, hypothesis 2 is also accepted. In other words, in the presence of anxiety, AAP more severely affects the employees’ performance Table 6.

**TABLE 6:** Indirect relationship.

Paths	Direct effect (*t*-value)	Indirect effect (*t*-value)	Total effect	VAF %	Interpretation	Results
AAP → Anty→EP	–0.173 (2.374)	–0.256 (4.613)	–0.428 (9.002)	59.81	Partial mediation	H2, supported

AAP, Ambient air pollution; Anty, anxiety; EP, employee performance.

## Discussion

The object of this study was to evaluate the impact of AAP on outdoor employees’ performance and examining the role of anxiety in the relationship between AAP and outdoor employees’ performance. The empirical pieces of evidence are collected from the pharmaceutical industry of Pakistan (outdoor employees/sales and marketing staff). The results of this study are very alarming and important for pharmaceutical firms and the outdoor employees of these firms. Drawn on the processing efficiency theory (Calvo and Eysenck, 1992), first, this study confirms the significant and negative relation between AAP and outdoor employees’ performance. Second, this paper also confirms the significant mediating role of anxiety between AAP and outdoor employees’ performance. Anxiety partially/complementary (VAF = 59.81%) mediates the above-said relationship (Hair et al., 2016). The results of this study agree with the results presented by Derakshan and Eysenck (2009), Ivandic et al. (2017), and Chang et al. (2019).

The AAP has become an open and global reality and its negative effects on humans and the mechanism of nature are very obvious. The employees who perform their duties outsides especially sales, marketing, and supply chain staff seriously face the impact of AAP. According to “The Global Competitiveness Report 2019” prepared by World Economic Forum, the countries (such as Singapore, United States, Hong Kong SAR, Netherlands, Switzerland, Japan, Germany, Sweden, United Kingdom, Denmark) have a better position in ecological footprint, enforcement of environment-related treaties, Social capital development have a better level of productivity and Gross domestic production (GDP) at individual and state levels, respectively. Contrary, the states (such as Bangladesh, Pakistan, Burundi, Chad, Afghanistan, Haiti, Mali, Mozambique, and Tajikistan) with a bad position in ecological footprint, enforcement of environment-related treaties, deficit in biocapacity, worst air pollution level have low productivity and GDP level at the individual as well as state level (World Economic Forum, 2019).

Globally, 1.2 billion workers spend their working hours outside. As per statistics of the World Health Organization (WHO), 4.2 million early deaths occur every year among the general population and a significant portion of these deaths belong to the outdoor employees (WHO, 2018). Outdoor Employees in the winter season are at higher risk of respiratory diseases, and the governments usually underestimate this sort of risk (Vinnikov et al., 2020). The workers are not much aware of the impact of AAP on their health while working outdoors. Therefore, it’s the responsibility of the organizations and the states to assist the employees (Vinnikov et al., 2020). British safety council has recommended some measures for outdoor employees (British Safety Council, 2020). For instance, the workers should reduce working time during rush hours and high pollution alerts. The job rotations and reduction in physical exertion at peak traffic hours also help to reduce the impact of AAP on outdoor employees. The pollution barriers and possible distance from the machinery are helpful to reduce the impact of AAP up to 20%. A good and balanced diet, a good quality face mask, and regular exercise help workers to fight against the effects of APP (British Safety Council, 2020).

The regular fluctuations in AAP did not affect the employees’ performance instantly. However, after 1-month exposures, the performance of employees will be dropped out (He et al., 2019). According to Prof. Liu, a rise in PM_2.5_ by ten micrograms/cubic meter continued over 25 days, the performance of workers and the organization will be decreased by 1% (He et al., 2019; Tabinda et al., 2019), some scholars claim workers output can decrease up to 6% (He et al., 2019). Although, the employees of the pharmaceutical industry are more informed and vigilant about AAP’s impact on their health and performance than the workers from other industries. However, outdoor staff (medical representatives) of pharmaceutical firms still face the impact of AAP because of intensive visits to doctors, long working hours, and regular long distance traveling (Harris et al., 2003). Therefore, pharmaceutical firms can improve performance of their outdoor employees by providing flexible working hours, arranging online meetings and presentations with customers and management, respectively, and providing safety material such as good quality face mask etc.

In this competitive era, organizations pay much money on their employees’ training to make them efficient. However, anxious employees do not give proper attention to their work, even not take interest in learning new skills. AAP not only a cause of physical diseases such as cardiovascular and respiratory disorders but also psychological problems such as anxiety and stress etc. One-month average of PM_2.5_ increases chances of high degree anxiety by 12% on every 10 μg/m^3^ increase (Power et al., 2015). The anxious workers underperform and usually miss assignment deadlines. Sometimes, anxious employees leave the job or develop some counterproductive work behaviors such as bad behaviors with peers and coworkers. To overcome the impact of AAP on employee’s health and performance, renewable energy and a green environment is a very effective solution. Therefore, ISO 14001 should be implemented in all organizations globally.

### Theoretical contribution

This study tested the processing efficiency theory (Calvo and Eysenck, 1992) while analyzing the relationship between AAP and employees’ performance. Furthermore, the role of anxiety between the negative relationship of AAP and employees’ performance. This study extended the literature on AAP and employees’ performance by contributing to the importance of anxiety between the above relationships. This is one of the first studies that tested the processing efficiency theory on employees of pharmaceutical firms in Pakistan. Hence, this study confirms the rationality of the processing efficiency theory in the Pakistani context and culture. Moreover, this study highlights the importance of outdoor employee’s health for organizational performance. In brief, empirical investigation of this study strength the processing efficiency theory by confirming that AAP impacts the employees’ health and performance, and because of AAP, anxiety develops in employees which further negatively impacts on employees’ health and performance.

### Managerial implications

This study also has some important implications for the individuals (outdoor employees), management of the pharmaceutical industry specifically, and other industries in general. First, outdoor employees must understand the toxic impact of AAP on their health and professional performance. The employees should take all necessary measures to avoid the impact of AAP such as regular wear good quality face masks, avoid to go out during peak traffic hours, regularly wash their hands and mouth, and take warm water/liquid food with short gaps. Second, sales and marketing are the driving force for any organization, if sales and marketing staff did not perform well organizations suffer significantly. Therefore, organizations should take care of their outdoor employees. Pharmaceutical firms should facilitate to their outdoor employees. For instance, free good quality face mask, comfortable vehicle, and allowances for long traveling, regularly arrange recreational activities, medical insurance, and job crafting facility. Third, the arrangements for an indoor green environment are also recommended to the managers, therefore, organizations should go for ISO 14001 certification to secure the green environment. Fourth, female workers are easily affected by AAP and anxiety than males (Harris et al., 2003). Thus, extra care of female employees from AAP by the firms is recommended. Fifth, the ministry of health can ask the pharmaceutical firms for ISO 14001 certification compulsory.

### Economic implications

Since AAP is an economic issue too. The high-level of AAP is not only increasing the health expenditure, but also lowering the performance of individuals, which in turn negatively impacting the organizational as well as economic growth of the country. The findings of this studies implies that by reducing the negative impacts of AAP on employees’ performance, organization can contribute the economic growth of the country. This is an important economic implication of this study.

## Conclusion

This study has examined the link between air pollution and outdoor employee performance work in pharmaceutical companies of Pakistan by considering the mediating role of anxiety. The findings show that air pollution is an important determinant of anxiety, which in turn decreases employees’ performance. This suggests that outdoor employees are at high risk of health deterioration that can significantly reduce their performance, hence the organizational performance. Therefore, it is essential for organizations take protection measure from the pollution so that employees’ anxiety can be decrease and performance can be increased.

As per the other studies of social sciences, this study also has numerous limitations and future research opportunities. First, this study is limited by its sample size which is just 299 and it is too small as compared with the total population. That’s why this study did not present a clear picture of all outdoor sales employees’ perceptions of working in pharmaceuticals companies in Pakistan. In the future, to confirm and strengthen the present study outcomes, a comparatively big sample size should be analyzed. Second, this study used various techniques to mitigate the problem of participant’s perception-based bias, however, perception bias may exist. In the future, some other techniques should be adopted such as experiential study. Third, the present study empirically investigated the outdoor sales employees of pharmaceutical companies in Pakistan. In the future, some other organizations can be considered with the same theoretical model such as transport, food delivery, restaurants, insurance, and banking sectors. Fourth, this study is conducted in a single culture, in the future, this theoretical model can be tested in cross-national cultures/organizational cultures or between developed and developing countries for a comparative study. Fifth, as per previous investigations (Power et al., 2015) the impact of AAP appears after 30 days. Therefore, a longitudinal study can provide better results. Finally, this study has collected on subjective data in air pollution, which can limit our insights in this important phenomenon. Future researchers are recommended to collect the secondary data too so the alternative insights can be gained.

## Data availability statement

The raw data supporting the conclusions of this article will be made available by the authors, without undue reservation.

## Author contributions

MoB: idea development, drafting and analysis. SS: drafting and support in data collection. BA: analysis, drafting, and editing. All authors contributed to the article and approved the submitted version.

## Footnotes

1 Roth, S. (2016). The contemporaneous effect of indoor air pollution on cognitive performance: evidence from the UK. Unpublished Manuscript. doi: 10.1017/CBO9781107415324.004

2 Fu, S., Viard, B., and Zhang, P. (2017). Air quality and manufacturing firm productivity: comprehensive evidence from China.
